# A Method for Accessing the Non-Slip Function of Socks in an Acute Maneuver

**DOI:** 10.3390/s23031378

**Published:** 2023-01-26

**Authors:** Dongwook Seo, Jinsu Eun, Yeonwoo Yu, Sangsoo Park, Kikwang Lee

**Affiliations:** 1 College of Physical Education, Kookmin University, Seoul 02707, Republic of Korea; 2 School of Global Sport Studies, Korea University Sejong Campus, Sejong 30019, Republic of Korea

**Keywords:** pressure sensors, non-slip function, dynamic motor task

## Abstract

The shoe upper hides the foot motion on the insole, so it has been challenging to measure the non-slip function of socks in a dynamic motor task. The study aimed to propose a method to estimate the non-slip function of socks in an acute maneuver. Participants performed a shuttle run task while wearing three types of socks with different frictional properties. The forces produced by foot movement on the upper during the task were measured by pressure sensors installed at the upper. A force platform was also used to measure the ground reaction force at the outsole and ground. Peak force and impulse values computed by using forces measured by the pressure sensors were significantly different between the sock conditions, while there were no such differences in those values computed by using ground reaction forces measured by a force platform. The results suggested that the non-slip function of socks could be quantified by measuring forces at the foot-upper interface. The method could be an affordable option to measure the non-slip function of socks with minimal effects from skin artifacts and shoe upper integrity.

## 1. Introduction

Measuring the non-slip function of socks is essential for providing guidance on what socks we should wear for injury prevention and performance enhancement. Socks could reduce the risk of blisters caused by repeated contacts during walking [1,2,3]. Especially in sports involving acute changes in movement direction (e.g., cutting, turning), performance is closely related to the foot’s relative motion to a contact area (e.g., shoe insole) within shoes [3,4], which was defined as slip in this study. Those movements often require ground reaction force (GRF) production in the medial-lateral direction, distinct from locomotor tasks producing GRF production mainly in the anterior-posterior direction [5]. Previous studies have used mechanical devices to estimate the static frictional properties of socks in the anterior-posterior direction in movements involving a small range of motion [6,7,8,9,10,11,12,13,14], which may not represent the GRF characteristics observed in acute maneuvers. Although the frictional properties of socks in acute maneuvers have been measured [4,15], it has still been challenging to measure the non-slip function of socks in such acute maneuvers because the foot motion is hidden by the shoe upper.

The invisibility of the foot-insole interface has limited the direct measurement of the non-slip function of socks in acute maneuvers. The non-slip function of socks has been measured by observing foot motion on an insole during acute maneuvers [4,16]. Those studies have made holes in the shoe upper or both the upper and socks and placed passive reflective markers directly on the foot skin that were tracked by a motion capture system. However, those results would be susceptible to measurement errors in marker position [4,16] and altered elastic properties of the shoe upper [17] caused by skin movement and holes, respectively. Although radiography technology has shown minimal skin artifact effects [18], radiation exposures could be a limiting factor for using that technique in general.

The kinetics of the foot within a shoe may be used to estimate the frictional properties of socks in acute maneuvers with minimal effects of skin artefact and minimal changes in the elasticity of the upper. The shoe upper of sport shoes has made of fabrics that are stretchable. The elasticity has enabled the foot to slip on a shoe insole during the acute movement [3], which could be caused by a significant shear force production in the opposite direction relative to the movement direction (e.g., medial-lateral direction). Observed in-shoe foot movements were around 10 mm during the braking phase in a 180° turn [4], suggesting that the in-shoe foot movement may result in a significant force production at the foot-upper contact area. To our best knowledge, however, research studies have not yet used forces produced at the foot against the shoe upper to estimate the non-slip function of socks.

Thus, the aim of this study was to develop a method for evaluating the non-slip function of socks in an acute movement. Fifteen healthy male volunteers performed a shuttle-run task while wearing socks with different frictional properties. Pressure sensors were attached to the inner part of the shoe upper, with which the fifth metatarsal made contact. Forces applied to those sensors were utilized to estimate the non-slip function of socks in the acute maneuver. Ground reaction force (GRF) was also measured by a force platform to examine the effects of socks on GRF production. We hypothesized that the method using forces measured by a pressure sensor installed to the upper can distinguish the friction characteristics of socks.

## 2. Materials and Methods

### 2.1. Participants

Fifteen male volunteers participated in the experiment. (Age: 25 ± 3 years, height: 174.5 ± 4.7 cm, mass: 71.2 ± 8.3 kg, mean ± SD). All participants wore US 9 size shoes and were instructed to tie their shoelaces with the strength of their usual sports activities. This study was approved by the institutional review board (IRB) of Kookmin university (IRB No. KMU-202111-HR288), and consent was obtained from each participant before participating in the experiment.

### 2.2. Types of Socks and Shoes

**Figure 1:**
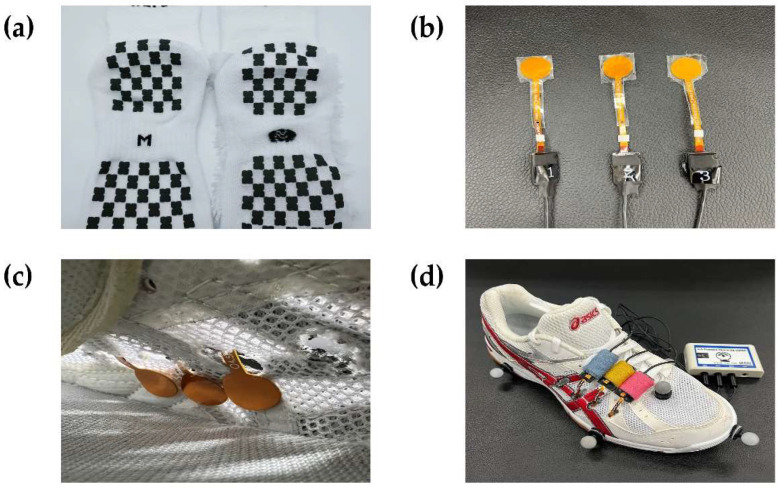
Socks, shoes, and equipment utilized in this study. (**a**) The US socks; (**b**) pressure sensors; (**c**) pressure sensors installed on the inner part of the shoe upper; (**d**) running shoes (Revele-FL5).

Three socks with different frictional properties were selected in this study [13]. Briefly, there were two types of non-slip socks: a polyurethane non-slip pad attached to both the inner and outer parts outside of socks (US; Figure 1a) and a silicone protection pad attached to the outside of socks (SS). The last one did not have any non-slip pads (NS). All socks had the same material, composition ratio, and shape except the non-slip pad (We Foot Technology, Gyeonggi-do, Republic of Korea). In this experiment, all subjects wore the same size shoes (ASCIS, Kobe, Japan). More details about the socks can be found elsewhere [13].

### 2.3. Emperimental Procedure

**Figure 2:**
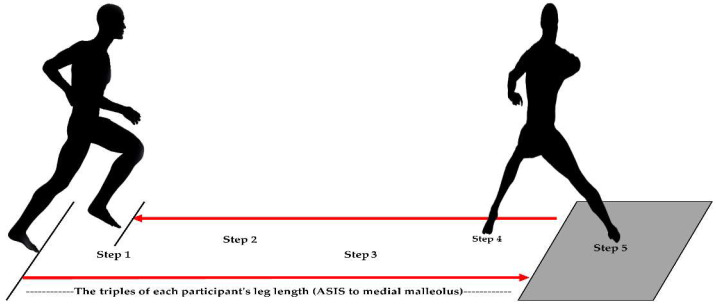
The shuttle run task utilized in this study [5,19]. The participants made a 180° directional change at the transition and came back to the starting position. They were instructed to perform the task as soon as possible.

The participants performed a shuttle-run task while wearing three different types of socks (US, SS, and NS). Each participant had a 10-min preparation session, including a warm-up and practice for a shuttle-run task to improve task familiarity. The shuttle-run task was initiated by making four steps in the forward direction, followed by a change of the movement direction to the opposite direction (180°) at the force platform and steps back to the starting point (Figure 2). Before each shuttle-run trial, standardized instruction was provided. They placed their feet before the starting point. Then, they initiated the task with a verbal sign (‘Go’) by running on the path of the red line, followed by placing their right foot at the right angle to the red line at the transition, and coming back to the starting position. They were instructed to complete the task as soon as possible. The distance between the starting and turning points was adjusted to the triple of each participant’s leg length (ASIS to medial malleolus). The participants performed five shuttle-run trials per each sock type, interspersed with a 2-min break to minimize fatigue.

### 2.4. Data Acquisition

Three pressure sensors (SEEDTECH, Gyeonggi-do, Korea, Figure 1b) were installed on the inner wall of a shoe (around the 5th metatarsal; Figure 1c) to measure force generated inside the shoe (Rote Rivre FL5, ASICS, Seoul, Korea; Figure 1d) during the shuttle-run trials. The pressure data were collected at 100 Hz via Bluetooth and then converted to force data in newtons (CubeFMS, SEEDTECH, Gyeonggi-do, Korea). The pressure sensor data were collected from all fifteen participants.

A force platform (AMTI, Watertown, MA, USA) was used to collect the ground reaction force data at 2000 Hz (Figure 2d). The ground reaction force data from four participants were excluded due to the sensor malfunction (*n* = 11). Thus, the ground reaction force data from the remaining eleven participants were utilized for further analyses.

The non-slip function of socks was quantified by computing slip time, maximum peak force, and impulse using the force data from pressure sensors (Python v3.9.5, Python Software Foundation, Wilmington, NC, USA). Those variables in the GRF data were computed by Visual 3D (Visual 3D v5™, C-Motion, Inc., Germantown, MY, USA).

**Figure 3:**
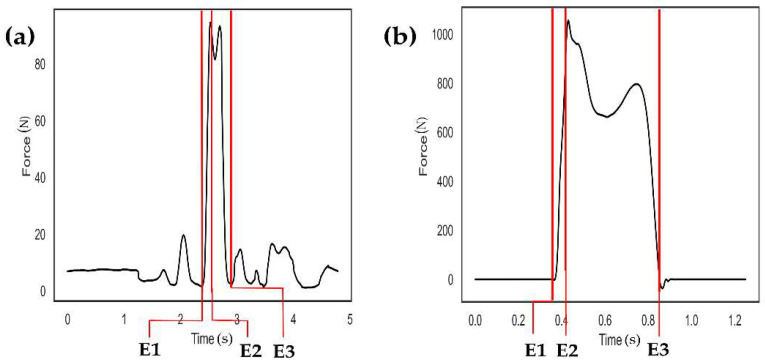
Three events were identified in the force data. (**a**) A representative force curve estimated by pressure sensors (black line) was shown. Event 1 (E1) was defined as the point at which the force value of the pressure sensor continued to increase due to the contact of the participant’s foot with the force plate, while Event 3 (E3) was defined as the time when the pressure value reached the lowest value. Event 2 (E2) was defined as when the pressure value reached the maximum between E1 and E3. (**b**) A representative force curve in the medial-lateral direction measured by the force platform (black line) was shown. Similarly, three events were identified as having been performed for the pressure sensor data.

There were events identified in the pressure and GRF data (Figure 3a,b). First, the on and off timings of the force data were identified (E1 and E3), followed by finding the first peak value (E2) during the on-off period. Slip time was computed by the difference between the on and off times, while impulse was computed by the integration of the force curves over the on-off period. The peak and impulse values were normalized by body weight.

### 2.5. Statistical Analysis

A one-way repeated measures ANOVA was performed to quantify differences in slip time, peak force, and impulse values between three sock types. When the sphericity assumption was violated, the Greenhouse–Geisser *p*-value adjustment was used. When the main effect was significant, pairwise comparisons with Bonferroni adjustments were performed. All statistical analyses were carried out using SPSS statistical software (V26, IBM, Armonk, NY, USA). Pearson’s correlation analysis was performed to evaluate whether there was a linear relationship between the variables computed using the pressure sensor data and the variables calculated using the GRF data. Pearson’s correlation coefficient r was defined as a weak positive linear relationship at 0.1 < r ≤ 0.3, a moderate positive linear relationship at 0.3 < r ≤ 0.7, and a strong positive linear relationship at 0.7 < r ≤ 1. The level of statistical significance was set at *p* < 0.05.

## 3. Results

**Figure 4:**
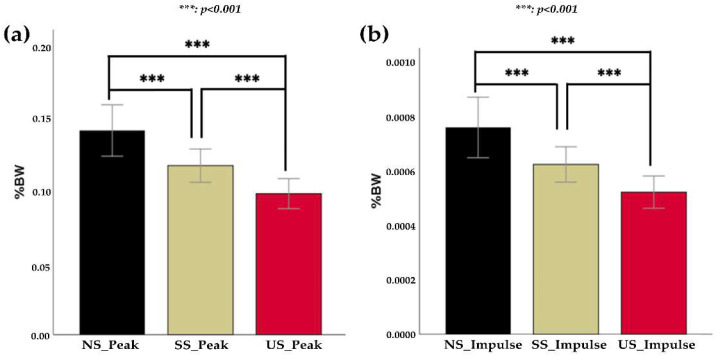
Comparison of peak and impulse values between socks. (**a**) Peak values (mean ± std) measured with three different socks (NS: normal socks, SS: silicone socks, and US: urethane socks) (**b**) Impulse values (mean ± std) measured with three different socks. Significant level: *** *p* < 0.001.

A repeated-measures ANOVA determined that mean peak force differed significantly between sock types (F (1.6,118.5) = 26.4, *p* < 0.001; Figure 4a). Post hoc analysis with Bonferroni adjustments found that peak force values were significantly greater in NS socks (0.1409 ± 0.07709) than in SS socks (0.1167 ± 0.04990, *p* < 0.001; Figure 4a) and in US socks (0.974 ± 0.04500, *p* < 0.001; Figure 4a). Peak force values from the NS sock were larger than peak force values from the US sock (*p* < 0.001). Similar results were found in impulse values, as indicated by a significant main effect (F (1.3,99.8) = 24.6, *p* < 0.001; Figure 4b), with significant differences in impulse values between NS (0.0008 ± 0.00048) and SS (0.0006 ± 0.00028, *p* < 0.001), between NS and US (0.0005 ± 0.00026, *p* < 0.001), and between SS and US (*p* < 0.001). However, there was no significant main effect of sock type on slip time (F (1.8,133) = 2.3, *p* = 0.105).

There were no significant differences between socks for peak values (F (2,108 = 2.5, *p* = 0.087), slip time values (F (2,108) = 2.2, *p* = 0.116), and impulse values (F (1.7,108) = 2.1, *p* = 0.132) computed from the GRF data. The correlation coefficient (r) values were summarized in Table 1. No significant correlation was found in all the comparisons except NS time.

## 4. Discussion

The non-slip function of socks in an acute maneuver was quantified by the force produced at the contact between the foot and shoe upper in this study. Peak force and impulse values were significantly different between socks, while such differences were not observed in those values estimated from the ground reaction force. The method suggested in this study successfully estimated the different frictional properties of socks, which the ground reaction force data could not measure.

### 4.1. Forces Applied to the Shoe Upper Can Be Used to Quantify the Non-Slip Function of Socks

Contact forces at the foot-upper interface could be used to measure the non-slip function of socks. Previous studies have shown greater foot motion on an insole with socks having lower friction [4,15], suggesting that the inner wall of the upper may experience contact force at the foot-upper interface. Based on the observation, the current study installed pressure sensors by minimally cutting the upper to measure the foot slip, which is not visible from the outside.

Different frictional properties of socks were successfully identified by the suggested method. Our earlier work found that the static coefficient of US socks was the highest compared to the other socks, followed by SS and NS [12], which was consistent with the non-slip function in the dynamic motor task. The US socks showed the smallest peak force and impulse values applied to the upper, suggesting that the socks showed the highest non-slip function. The largest peak force and impulse values were observed in the NS socks that did not have any additional protrusions or non-slip materials attached. These results suggested that sock designs and materials could affect the non-slip function of socks in a dynamic task.

The observed changes with sock types were not found in slip time, maximum peak force, and impulse values identified in ground reaction force (GRF) data, which was consistent with the observations in a previous study [4]. Further, those values were not significantly related to those values computed by using the pressure sensor data in general. The observed discrepancies between the in-shoe and out-shoe forces would be explained by the forces each piece of equipment measured during the shuttle-run task. The force platform measured forces produced at the outsole-ground contact. With the assumption that the force dissipation occurred at the midsole was minimal, the forces may result from the summation of frictional forces at the foot-insole and forces at the foot-upper contact, of which the letter one was measured by the pressure sensors. That is, the sensors at the upper level measured the sub-component of the resultant GRF only, leading to discrepancies in findings between the force platform and pressure sensors. These interpretations suggest that the foot slip occurring within a shoe could be better captured by measuring the force applied to the upper than GRF data.

### 4.2. Using Pressure Sensors May Be an Affordable Option to Measure the Non-Slip Function of Socks with Less Effects of Skin Movement and Upper Elasticity Changes

The method suggested in this study may be a cost-effective option that minimizes the effects of skin artifacts and changes in shoe integrity. Previous studies have made holes in the shoe upper to observe foot motion inside shoes [4,15], which may affect the shoe elasticity [17]. The altered elasticity would affect the amounts of foot slip during acute maneuvers so that it would be unable to measure the non-slip function of socks in an intact shoe condition. Further, the foot motion in the earlier studies was quantified by the position of passive markers directly attached to the foot skin. Skin movements under the markers may also affect the measurement of foot motion, while using a motion capture system could also be expensive. In the current study, we cut the shoe upper minimally to attach pressure sensors to the inner part that the foot makes contact with. The installation eliminated the need for marker placement and making several holes in the upper, leading to reduced effects of the skin artifacts and changes in upper shoe properties on the frictional property measurement in acute maneuvers. Due to its portability, the measurement system utilized in this study could be used outside the laboratory, including in sporting fields.

### 4.3. Study Limitations and Future Directions

There were several limitations in this study. First, there would be the learning effects on participants during repeated trials, although we randomized the order of socks to minimize the learning effects. Strategies to perform and adapt to the shuttle run task may differ between individuals and sock conditions. Future studies investigating the strategy may provide insight into the effects of socks with different frictional properties on performance and adaptation. Secondly, the different foot shapes of individual participants were not considered. The contact area of the foot with pressure sensors may differ among participants due to their different foot shapes. Although three pressure sensors rather than one were attached near the fifth metatarsal to reflect the shape difference, there would be differences in the foot contact area between participants. Lastly, the findings in the current study may not be generalizable to the other gender. Although a previous study reported differences in foot motion during acute maneuvers between males and females, those differences were not affected by different sock types [4], suggesting that the findings in the current study may not be affected by gender. Future studies with both genders are needed to confirm the interpretation.

## 5. Conclusions

The non-slip function of socks in an acute maneuver was measured by pressure sensors installed at the shoe upper. Peak force and impulse values were significantly different between socks, while such differences were not observed in those values estimated from the ground reaction force. The method suggested in this study successfully estimated the different frictional properties of socks with minimal effects on skin artifacts and shoe integrity. The suggested method could be an affordable option and be performed outside of the laboratory.

## Figures and Tables

**Table 1:** Pearson’s correlation values for slip time, peak, and impulse values between pressure sensor data and GRF data (NS: normal socks, SS: silicone socks, and US: urethane socks; r: correlation coefficient; **: *p* < 0.05).

	NS	SS	US
Time	r = 0.301	r = 0.002	r = −0.045
	*p* = 0.025 **	*p* = 0.989	*p* = 0.746
Peak	r = 0.121	r = 0.057	r = −0.133
	*p* = 0.377	*p* = 0.679	*p* = 0.331
Impulse	r = −0.46	r = −0.179	r = 0.025
	*p* = 0.739	*p* = 0.191	*p* = 0.854

## Data Availability

The data utilized in this study is available upon request.
